# Dropping bad habits

**DOI:** 10.2471/BLT.21.021021

**Published:** 2021-10-01

## Abstract

Robust enforcement of tobacco control legislation is helping curb tobacco use in the Russian Federation. Andrey Shukshin reports.

When, in 2017, 45-year-old Alexey Semenov (name changed at his request) applied for a welder’s job at a firm located just outside Moscow in the Russian Federation, he barely paid attention to the words “No Bad Habits” that were underlined on the application form, considering it a stock phrase used by employers to discourage applicants dependent on alcohol. But at the interview for the job, he received a surprise. “It turned out that bad habits included tobacco use,” he says.

The company, a contractor with the defence ministry, had made tobacco control a matter of corporate policy. Having denied any such habits, Alexey got the job. Then came the challenging task of making his denial true.

That shift in corporate policy reflects a broad transformation in the Russian Federation’s tolerance for tobacco use which has its origins in the country’s accession to the World Health Organization (WHO) Framework Convention on Tobacco Control (FCTC) in 2008.

According to Viktor Zykov, a tobacco control expert at the State Research Institute for Health Organization and Informatics of the Ministry of Health of the Russian Federation and member of the Coordination Council for Tobacco Control, the Russian Federation joined the FCTC (an instrument which the Russian Federation helped to develop) in response to steadily worsening tobacco use trends in the 1990s and early 2000s.

Those trends were driven by the low cost and wide availability of cigarettes, the lack of restrictions on smoking and tobacco advertising, and the aggressive marketing of brands by tobacco companies, including multinationals that expanded into the Russian Federation market in the 1990s after the collapse of the Soviet Union. “Tobacco use prevalence at the time of joining the FCTC was catastrophic,” Zykov says. "By 2009, almost four in every 10 adults smoked – a dreadful figure."

The country’s accession to the FCTC was followed by the passage of robust anti-tobacco legislation in June 2014. One of the toughest provisions of the law was the partial, and then – from 1 January 2015 – a complete ban on smoking in public places. The sale of tobacco products near educational institutions and on the internet was also banned, as was the advertising of tobacco products, and their promotion in the media.

Since 2017, 50% of the surface area of cigarette packages must carry warnings about the dangers of smoking and graphic images of the consequences of diseases caused by tobacco. The law also provides for a gradual increase in tobacco excise duty and the organization of medical and other assistance for people wishing to quit.

One of the more controversial aspects of the new regulations was the ban on tobacco sales in tobacco stalls and kiosks, which, prior to 1 June 2014, could be found at almost every public transport stop. On that date, and almost overnight, those outlets disappeared or stopped selling tobacco products, making it harder for smokers to get hold of cigarettes.

“Tobacco companies around the world fought tooth and nail.”

Victor Zykov

Critics of the measure argued that it stifled small business and was implemented without regard to public opinion. “The opposition was enormous, and included outside support,” Zykov recalls, noting the efforts made by multinational tobacco companies to push back against the measure. “Tobacco companies around the world fought tooth and nail, employing a variety of tactics,” he says.

Despite such resistance, the public health authorities have not wavered in enforcing the law, backed by the president. “President Putin really got behind the law,” Zykov says. “As a result, tobacco control measures have been implemented rigorously.”

Leadership from the top has been complemented by significant grass roots support, notably support mobilized through the Ministry of Health's “No Smoking Here” app, which is designed to help people report violations of the law. According to Zykov, tens of thousands of fines have been imposed on people violating laws, many of them denounced via the app.

Estimates vary as to the impact of these measures, but all indicate significant downward trends. According to WHO’s Global Adult Tobacco Survey tobacco use prevalence among the adult (15+) population fell from 39% (44 of 112 million) in 2009 to 30% (36 of 120 million) in 2016. The Russian Public Opinion Research Centre reports a similar drop but over a longer period, with estimated tobacco use prevalence among the adult population falling from around 42% to 28% between 2009 and 2020. Meanwhile, the Federal State Statistics Service reports an estimated decline in prevalence from 34% to 22% over the same period.

On World No Tobacco Day in June 2021, WHO officially recognized the Russian Federation's achievements as "outstanding", noting, among other things, that the government's efforts had helped to reduce tobacco sales by around a third in just seven years, from 2009 to 2016.

For Dr Vinayak Prasad, programme manager of WHO’s Tobacco Free Initiative, the Russian Federation’s significant achievements constitute a solid foundation on which to build.

“WHO's Report on the Global Tobacco Epidemic 2021 found that the Russian Federation has scope for progress on two of the six most important indicators by which WHO measures countries' progress – increasing tobacco excise duty and cessation support,” he says.

Raising taxes to make tobacco less affordable has been shown to discourage not only users but also those thinking of starting. It is thus crucial in preventing adolescents from taking up the habit. “Preventing youth initiation is key to avoiding the creation of a new generation of tobacco users,” Prasad says, pointing out that the Global Youth Tobacco Survey conducted in Moscow in 2015 showed that 14.1% of children aged 13–15 were already using tobacco products.

As regards excise duty, the finance ministry reports that the minimum excise rate has already risen 23 times since 2007, driving significant increases in retail prices. Tax as a share of the most popular brand’s retail price stood at 56.1% in 2020, moving towards WHO’s best practice level of 75%. In 2021, the minimum excise rate has jumped by a further 20%, but according to a finance ministry statement this was a one-off increase to generate additional revenue for the pandemic-hit federal budget. Going forward, annual rises are expected to track inflation, a rate of increase judged by Zykov to be insufficient.

With regard to cessation support, there is also potential for further measures. According to Prasad, “Help to quit” services are subsidized and available in some health care facilities in the country, but not everywhere, a fact lamented by Dr Olga Sukhovskaya, a tobacco control expert, who heads up the national Tobacco Cessation Call Centre in Saint Petersburg.

“Less than 1% quit smoking for economic reasons in 2012. Now it’s closer to 25%.”

Olga Sukhovskaya

“The 2014 law and the recently adopted Clinical Practice Guidelines require that every doctor should know how to treat nicotine addiction, be able to prescribe treatment and give basic advice, but more specialized training is needed, so that doctors can provide real help rather than just say ‘Stop smoking!’, which, unfortunately, still happens,” she says.

The establishment of the national cessation centre in 2011 is something of a success story, and the centre’s visibility increased significantly in 2018, when its phone number started to appear on every pack of cigarettes sold.

In addition to providing counselling and sharing information about medical institutions and medicines available to help users quit, the centre also presents researchers and policy-makers with informal but suggestive insights into the way the tobacco use landscape may be evolving.

When it comes to smokers’ awareness regarding cessation support medication, for example, Sukhovskaya reports that in 2013 around 4% of people contacting the centre were aware of the existence of such medicines. “Now it is more like 20%,” she says. There are also signs that tobacco users are becoming more sensitive to price. “In 2012 around 1% of people who quit smoking did so for economic reasons. In 2021, the figure is closer to 25%,” she says.

Going forward, novel nicotine and tobacco products are likely to present a major challenge. The above-cited Global Youth Tobacco Survey conducted in Moscow in 2015 showed that 14.5% of children aged 13–15 were already using electronic cigarettes and the number has likely grown since then.

Last year, the Russian Federation passed amendments to the 2013 anti-tobacco law that equated new nicotine-containing products with conventional ones, imposing bans as appropriate – including bans on advertising and promotion.

“The latest amendments to the law pave the way for further advances,” says Sukhovskaya. Zykov, who hopes to keep the share of such devices in the marketplace to the minimum, agrees, considering the changes to the law to be “a very big step forward.”

For Alexey Semenov, the big step forward was getting off the tobacco treadmill altogether. It wasn’t higher prices that stopped him or dissuasive packaging or even his company’s anti-smoking policy. It was the novel coronavirus disease 2019. 

Semenov spent several weeks in a Moscow hospital in March 2020 with a severe form of COVID-19. “I stopped after that,” he says. “I still have some bad habits, but smoking isn’t one of them.”

**Figure:**
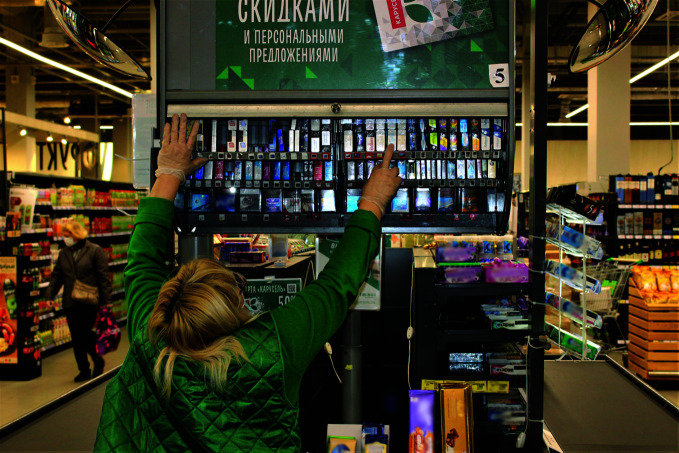
Opaque cabinet opened to access tobacco products in Moscow retail outlet.

Courtesy of Andrey Shukshin

**Figure:**
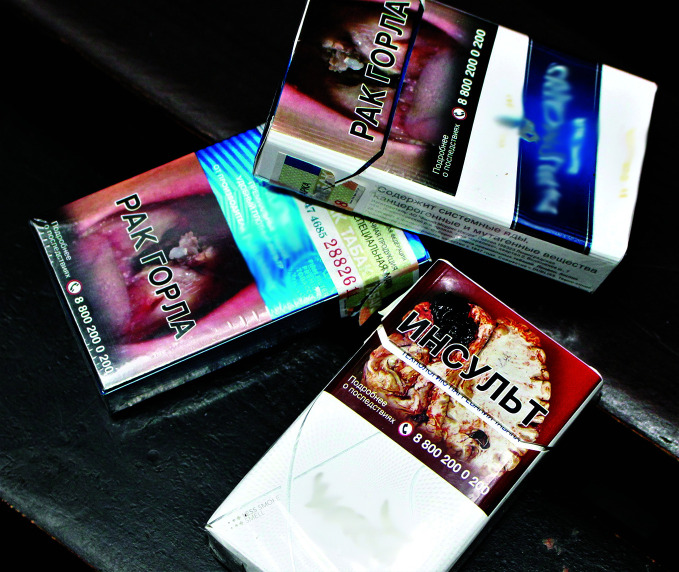
Cigarette packaging with health warnings and dissuasive images.

Courtesy of Andrey Shukshin

